# Guidelines on Immunization of People with Chronic Neurological Disorders: A Systematic Review

**DOI:** 10.3390/vaccines14090777

**Published:** 2026-09-05

**Authors:** Giuseppina Lo Moro, Andreea Mihaela Butnaru, Carola Toffanin, Anastasia Cataldo, Simona Mazzeo, Manuela Martella, Giacomo Scaioli, Fabrizio Bert, Roberta Siliquini

**Affiliations:** 1 Department of Public Health and Pediatric Sciences, University of Turin, 10126 Torino, Italy; giuseppina.lomoro@unito.it (G.L.M.); andreeamihaela.butnaru@unito.it (A.M.B.); simona.mazzeo@unito.it (S.M.); manuela.martella@unito.it (M.M.); giacomo.scaioli@unito.it (G.S.); fabrizio.bert@unito.it (F.B.); roberta.siliquini@unito.it (R.S.); 2 University Hospital “Azienda Ospedaliero-Universitaria” A.O.U. City of Health and Science of Torino, 10126 Torino, Italy

**Keywords:** immunization, vaccination guidelines, chronic neurological disorders, neurological diseases, vaccine-preventable infections, clinical practice guidelines

## Abstract

Background: People with chronic neurological disorders may be vulnerable to severe outcomes from vaccine-preventable infections. However, immunization recommendations for these patients may be difficult to identify and apply. This systematic review aimed to identify and describe official guidelines on preventive active immunization for individuals with chronic neurological disorders. Methods: This systematic review was conducted according to Preferred Reporting Items for Systematic Reviews and Meta-Analyses (PRISMA) 2020 and registered in the PROSPERO International prospective register of systematic reviews (CRD420251005848). Bibliographic databases (PubMed, Embase, Scopus) and grey literature sources were searched to identify official national or international clinical practice guidelines or comparable structured guidance documents providing recommendations relevant to chronic neurological disorders. The search was restricted to countries with a Human Development Index greater than 0.8, to allow comparison across broadly comparable healthcare and socioeconomic contexts. Data were extracted on document characteristics, neurological conditions addressed, vaccines covered, and vaccination-related recommendations. Results: Overall, 12 unique documents were included. Four were vaccination-focused documents including recommendations for neurological disorders, four were disease-management guidelines including vaccination-related recommendations, and four specifically addressed vaccination in selected neurological diseases. Guidance was unevenly distributed across countries, neurological conditions, and document types. Influenza and COVID-19 vaccination were the most consistently addressed vaccines. Multiple sclerosis was the condition with the most detailed guidance, especially regarding vaccination before disease-modifying therapies, live attenuated vaccines, vaccine timing in relation to immunosuppression or relapse, and protection of close contacts. Conclusions: Official immunization guidance for people with chronic neurological disorders remains limited and heterogeneous. Future guidelines should provide clearer and regularly updated recommendations to support vaccination assessment, treatment-sensitive planning, and multidisciplinary coordination.

## 1. Introduction

### 1.1. Rationale

Neurological disorders are among the leading causes of disability and mortality worldwide, and their burden is increasing because of population growth and population ageing [1]. Although chronic neurological disorders represent a heterogeneous group of conditions, several clinical features may increase vulnerability to vaccine-preventable infections or to their complications.

This vulnerability may be driven by different mechanisms. In some neurological and neuromuscular conditions, impaired respiratory muscle strength, reduced cough effectiveness, dysphagia, aspiration risk, reduced mobility, or compromised pulmonary function may increase the risk of severe respiratory outcomes after common infections such as influenza or pneumococcal disease [2]. In other conditions, the risk may be related to the use of immunomodulatory or immunosuppressive therapies. These treatments can increase susceptibility to infections, reduce vaccine-induced immune responses, and affect the safety of live attenuated vaccines, making vaccination timing an important component of treatment planning [3].

Despite the clinical relevance of vaccination in these populations, vaccine uptake may remain suboptimal. Studies in selected neurological populations, including people with multiple sclerosis, children with epilepsy, and children with underlying neurological conditions, have reported delayed or missed immunization, concerns about vaccine safety, fear of disease worsening, and uncertainty about interactions between vaccines and neurological treatments [4,5,6,7]. These findings suggest that vaccine recommendations for neurological patients need to be clear and accessible.

Healthcare professionals have a central role in addressing these gaps. Evidence from neurological settings indicates that vaccine recommendations may vary across practitioners, e.g., for conditions such as myasthenia gravis or chronic inflammatory demyelinating polyneuropathy, and that clearer professional guidance and education are perceived as unmet needs [8]. In people with multiple sclerosis, personal communication with healthcare professionals and tailored information on disease-modifying therapies, vaccine safety, and expected side effects have been identified as important elements to address vaccine hesitancy [9]. Similarly, gaps in awareness of existing guidance among neuromuscular neurologists highlight the need for neurology-specific and operational recommendations when vaccination is relevant to treatment safety, such as before chronic glucocorticoid therapy [10].

For these reasons, official guidelines may support safer and more consistent immunization practices in people with chronic neurological disorders. However, it is unclear which national or international guidance documents are available, which neurological conditions they address, and how vaccination recommendations are formulated across countries and document types. 

### 1.2. Objectives

This systematic review aimed to identify and describe official national and international guidelines addressing preventive active immunization for individuals with chronic neurological disorders across selected countries with broadly comparable healthcare and socioeconomic contexts.

## 2. Materials and Methods

This systematic review was conducted following the PRISMA 2020 guideline and checklist [11]. The review protocol was registered on PROSPERO (CRD420251005848). The main review question was “What is the current state of the art about published official national and/or international vaccine guidelines for patients affected by chronic neurological disorders?”.

### 2.1. Eligibility Criteria

The search and inclusion criteria were guided by the Population, Intervention or Phenomena of Interest, Context (PICo) framework [12].

Population target included adult and pediatric patients affected with chronic neurological disorders, whereas exclusion criteria concerned general population, people affected with non-neurological diseases, or acute neurological disorders except for stroke and its sequelae. Chronic neurological disorders were included based on the International Classification of Diseases, 11th Revision (ICD-11) [13] and are detailed in the Supplementary Material (Supplementary Section S1).

The Phenomenon of Interest was represented by official clinical practice guidelines addressing preventive immunization against infectious diseases. Guidelines were eligible if they addressed active immunization as a preventive measure against infectious diseases in adults or children with chronic neurological disorders. Therapeutic vaccines were outside the scope of the review and were therefore excluded. Clinical practice guidelines were defined according to the Institute of Medicine (IOM) as “statements that include recommendations, intended to optimize patient care, that are informed by a systematic review of evidence and an assessment of the benefits and harms of alternative care options” [14]. Only guidelines issued at national or international level were eligible. Regional, local, hospital-based, or single-center protocols were excluded. Documents were retained for the synthesis when they were issued, endorsed, or formally supported by a national or international institution, public health authority, or scientific society. National immunization handbooks or programme documents were considered eligible when they provided evidence-based recommendations across the immunization schedule, reported sufficiently transparent methods for evidence review and recommendation development to be considered comparable to clinical practice guidelines, and included specific sections or risk-group recommendations relevant to chronic neurological disorders. In addition to guidelines, scientific articles reporting official guidelines that met the above-mentioned criteria were included. Documents based solely on consensus conferences, expert opinion, or practical recommendations without a clearly reported evidence-review and recommendation-development process were excluded from the synthesis. Conversely, documents labelled as consensus statements, consensus recommendations, or care considerations were considered eligible when they provided explicit clinical recommendations and reported a structured evidence-review and recommendation-development process consistent with the predefined definition of clinical practice guidelines.

The Context was restricted to countries with a Human Development Index (HDI) greater than 0.8. HDI is a composite measure of social and economic development, calculated as the geometric mean of normalized indices for health (life expectancy at birth), education (mean years of schooling), and economic status (Gross National Income per capita). Countries exceeding this threshold generally have high levels of health, education, and income indicators [15]. The list of countries is provided in Supplementary Material (Supplementary Section S2). This criterion was adopted as a pragmatic way to delimit the scope of the review and to focus on countries with broadly comparable levels of human development and resource availability. However, HDI was not intended to represent the full complexity of national healthcare and immunization systems, which may vary substantially even among countries with similar HDI values.

### 2.2. Information Sources and Search Strategy

A comprehensive search strategy was applied to both scientific bibliographic databases and grey literature sources. A 10-year publication period, from 2014 to 2024, was considered appropriate to capture contemporary vaccination guidelines while minimizing the inclusion of outdated recommendations. Languages selected were English, Italian, French, German, Spanish, Romanian, Portuguese, i.e., the languages known to the Authors. 

Grey literature was searched systematically on a country-by-country basis between 1 March and 1 July 2024. For each eligible country, institutional and governmental websites, including Ministries of Health and national public health institutes, were screened to identify official documents outside academic publishing channels. Websites of relevant national scientific or medical societies were also searched when applicable. Searches were performed using predefined combinations of terms related to guidelines, neurological disorders, and vaccination, adapted to the language and country of interest. Website search bars, Google searches, and relevant website sections on vaccination, immunization, neurological disorders, and clinical guidelines were screened. Searches were conducted both including and excluding COVID-19-related results, using the term “-COVID”, because preliminary searches showed that COVID-19-related documents dominated the results and could obscure other relevant vaccination guidelines. Additional international organizations were considered when directly linked from the official websites searched.

The bibliographic search was conducted in PubMed/MEDLINE, EMBASE, and Scopus on 2 September 2024. The search strategy combined three main concepts: guidelines, neurological disorders, and vaccination. Search terms within each concept were combined using the Boolean operator “OR”, and the three concepts were combined using “AND”. No “NOT” operator was used in the bibliographic database searches. The PubMed/MEDLINE strategy included both free-text terms and Medical Subject Headings (MeSH), while the Embase strategy included both free-text terms and Emtree subject headings. The Scopus strategy used free-text terms and indexed terms where available. The complete search strategy for each database is provided in Supplementary Material (Supplementary Section S3).

For records retrieved from bibliographic databases and assessed in full text, reference lists were screened to identify additional potentially eligible guidelines. Furthermore, when a guideline was identified, the issuing organization’s website and related official sources were checked to ascertain whether a more recent version or update was available. When multiple documents from the same issuing body addressed overlapping recommendations, the most recent document was retained for the synthesis.

### 2.3. Selection Process

Results from scientific literature search were screened by independent, blinded reviewers using a two-stage process. First, titles and abstracts were assessed against the inclusion and exclusion criteria; a pilot test on the 5% of records was done to test the inclusion and exclusion criteria. Subsequently, the full texts of potentially relevant articles were reviewed. Screening was performed in Rayyan [16]. Discrepancies were resolved through discussion.

Grey literature was assessed by independent reviewers, with allocation based on their language proficiency. When more versions have been found, only the most up-to-date documents have been considered.

### 2.4. Data Collection Process and Data Items

Data extraction was performed independently by two reviewers using a predefined extraction form. Disagreements were resolved by discussion or, when necessary, by consultation with a third reviewer. For each eligible guideline, we extracted title, issuing organization, country or geographical scope, year of publication or update, document type, target population, neurological condition(s) addressed, vaccine(s) or immunization topics covered, and whether the document was primarily vaccine-focused or disease-focused. Vaccination recommendations relevant to people with chronic neurological disorders were extracted, including routine immunization, vaccine-specific indications, contraindications and precautions, timing in relation to disease activity or immunosuppressive/immunomodulatory treatment. 

### 2.5. Study Risk of Bias Assessment

The quality of the included guidelines was appraised using the Appraisal of Guidelines for Research and Evaluation (AGREE II) instrument [17]. AGREE II scores were used descriptively and were not used as exclusion criteria. 

### 2.6. Effect Measures

No effect measures were calculated, as this review did not aim to estimate intervention effects or compare vaccine effectiveness or safety outcomes across studies. The objective was to identify and describe official guidance documents and to narratively synthesise their recommendations. Therefore, no summary effect estimates, measures of association, or comparative effect measures were applicable.

### 2.7. Synthesis Methods

Recommendations were narratively synthesized and organized into summary tables. Guidelines were grouped according to their primary focus: national immunization handbooks or programme documents, disease-specific neurological guidelines including vaccination-related recommendations, and guidelines specifically addressing vaccination in specific diseases. For national immunization guidelines, recommendations were summarized by vaccine or recommendation area. Disease-specific neurological guidelines were synthesized by neurological condition. 

### 2.8. Reporting Bias Assessment, Certainty Assessment

Reporting bias and certainty of evidence assessments were not applicable because this review synthesised official guideline documents rather than effect estimates from primary studies. No meta-analysis was performed. 

## 3. Results

### 3.1. Study Selection

**Figure 1:**
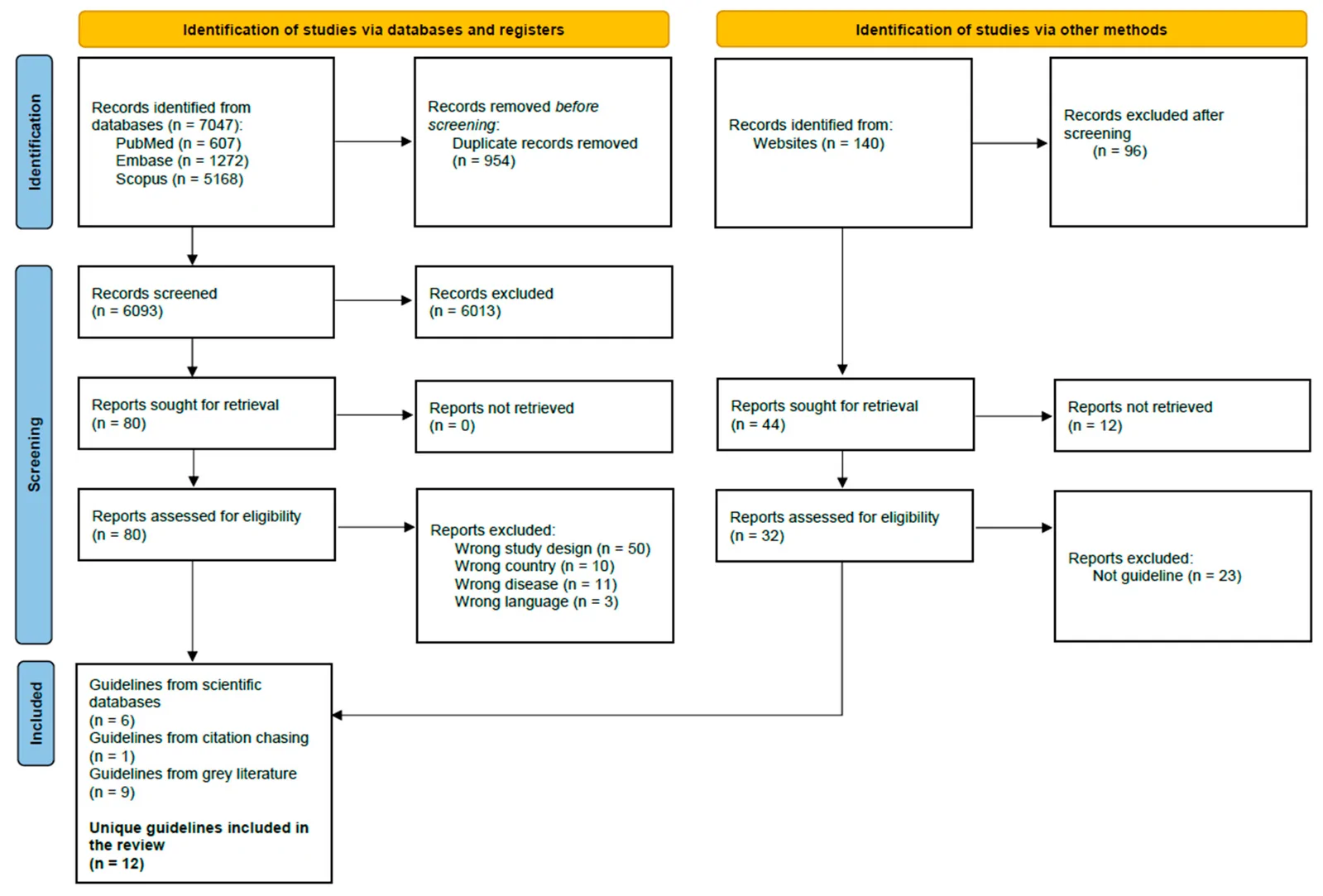
Flow diagram.

The grey literature search identified 140 documents from institutional and governmental sources. Of these, 44 were considered potentially relevant and 32 were retrieved and assessed in full text. Twenty-three documents were excluded because they did not meet the predefined definition of formal clinical practice guidelines, and nine documents were selected [18,19,20,21,22,23,24,25,26]. The bibliographic database search yielded 7047 records. After duplicate removal, 6093 records were screened. Six papers met the inclusion criteria [22,26,27,28,29,30] and one was retrieved through citation chasing [31]. Two papers reported guidelines already identified through the grey literature search [22,26]. Among the documents, three publications issued by the Société Francophone de la Sclérose en Plaques and the French Group for Recommendations in Multiple Sclerosis (SFSEP/France4MS) were identified [22,27,30]: the 2019 recommendations on immunization and multiple sclerosis [22], the 2021 recommendations on infections and multiple sclerosis [30], and the 2024 update on vaccination and multiple sclerosis [27]. Since the 2024 document represented the most recent vaccination-specific update, it was retained for the synthesis [27]. Therefore, 12 unique documents were included in the synthesis. The PRISMA flow diagram summarizing the identification and screening process is presented in Figure 1 [11].

### 3.2. Study Characteristics

The included guidelines originated from different geographical areas: Europe/UK (*n* = 5) [23,24,25,27,28], North America (*n* = 3) [19,26,31], Oceania (*n* = 2) [18,20], South America (*n* = 1) [29], and Asia (*n* = 1) [21]. Four guidelines defined national immunization programmes and included specific information for patients with chronic neurological disorders [18,19,20,21]. Four guidelines focused on the clinical management of specific neurological conditions and included dedicated sections on vaccination, namely multiple sclerosis (MS) [24,25], cerebral palsy [23], and Duchenne muscular dystrophy [31]. Finally, four guidelines specifically addressed vaccination in patients with multiple sclerosis [26,27,28,29] (Table 1).

### 3.3. Risk of Bias in Studies

AGREE II appraisal showed that all twelve included guidelines were suitable for use, although methodological quality varied across documents (Supplementary Section S4). Overall quality was high for most guidelines, with the two NICE guidelines receiving the highest rating [23,24]. The highest and most consistent scores were observed for scope and purpose and clarity of presentation, whereas the greatest variability was found in applicability and editorial independence, mainly because implementation barriers, resource implications, audit criteria, funding influence, and conflict-of-interest management were not consistently reported. Rigor of development was strongest for the NICE and Italian guidelines [23,24,25], the ECTRIMS/EAN consensus [28], and the US multiple sclerosis vaccination guideline [26], whereas it was lower in some handbooks, where systematic evidence selection and updating methods were less clearly described.

### 3.4. Results of Individual Studies

#### 3.4.1. National Immunization Guidelines Including Recommendations for Neurological Conditions

Four national immunization guidelines included recommendations relevant to people with neurological conditions [18,19,20,21]. (Table 2) Only one of these documents contained a section exclusively dedicated to neurological patients [19]; in the remaining guidelines, neurological conditions were addressed within vaccine-specific recommendations or risk-group indications. All four guidelines addressed both pediatric and adult populations.

Overall, they consistently indicated that routine vaccination can be performed in individuals with stable neurological conditions, provided that no other vaccine-specific contraindications are present. For vaccines other than those mentioned below, the guidelines did not provide additional recommendations specific to neurological conditions, but referred to the routine immunization schedule in general.

Pertussis-containing vaccines represented the main area in which neurological-specific precautions or contraindications were reported; however, recommendations were not fully uniform across documents. One guideline advised postponing pertussis vaccination in the presence of progressive or uncontrolled neurological disorders, such as severe uncontrolled epilepsy or progressive encephalopathy [18]. By contrast, another guideline supported pertussis vaccination even in children with unstable neurological conditions, provided that cases are assessed individually, because these children may be particularly vulnerable to severe pertussis complications [20]. A further guideline identified specific neurological events after previous vaccination, including encephalopathy of unknown cause occurring shortly after a pertussis-containing vaccine, as contraindications to subsequent Tdap administration [21].

Beyond routine schedules, all four guidelines recommended influenza vaccination for people with neuromuscular or central nervous system conditions, reflecting their increased risk of complications from influenza [18,19,20,21]. One guideline also highlighted COVID-19 vaccination for a broad range of neurological conditions, including stroke, neurodegenerative diseases, myasthenia gravis, multiple sclerosis, cerebral palsy, myopathies, paralytic syndromes, and epilepsy [20].

Pneumococcal vaccination was specifically addressed in relation to neurological conditions in two guidelines [19,20]. One of these provided detailed risk-based indications, recommending pneumococcal vaccination for individuals with cerebrospinal fluid leak, for children and older adults with neurological conditions, and for adults with neurological disorders that impair clearance of oral secretions, based on clinical judgement [19].

While recommendations for immunocompromised individuals were addressed more broadly across the guidelines [18,20,21], only one guideline explicitly related them to neurological disease management, noting that some conditions, such as multiple sclerosis, may be treated with immunosuppressive therapies. In these cases, general recommendations for immunocompromised individuals should be followed, including contraindications to live vaccines when applicable [19].

#### 3.4.2. Disease-Specific Neurological Guidelines Including Vaccination Recommendations

Eight guidelines focused on specific neurological conditions and included vaccination-related recommendations [23,24,25,26,27,28,29,31]. Three guidelines were restricted to adult patients [23,24,25].

Among disease-specific neurological guidelines, most recommendations concerned people with MS (Table 3).

Routine vaccination according to the general population schedule was generally considered appropriate, unless specific contraindications were present [26,27,28,29]. A recurrent recommendation was to assess and update vaccination status early in the disease course and before starting disease-modifying, immunosuppressive, immunomodulatory, or high-efficacy therapies, in order to reduce infection risk and avoid delays in treatment initiation [25,26,27,28,29]. This preventive approach was supported by the observation that MS itself does not cause immunosuppression, whereas infection risk may increase indirectly because of disease-modifying therapies or disease-related complications [29]. Some guidance provided more operational indications, including vaccination assessment at least 4–6 weeks before immunosuppressive or immunomodulatory therapy [26] and evaluation of previous infectious diseases and vaccination needs, preferably with infectious disease or public health specialists, before high-efficacy treatments [25].

Recommendations on live attenuated vaccines were broadly consistent across MS guidelines. These vaccines were generally contraindicated or should be avoided during treatment with immunosuppressive or selected disease-modifying therapies and after recent treatment discontinuation [25,26,27,28,29]. Conversely, live attenuated vaccines were considered usable in untreated patients or in those receiving lower-risk immunomodulatory treatments, such as interferon beta or glatiramer acetate [28]. Across the guidelines, treatment-specific indications, contraindications, and timing considerations were provided for several therapies, including high-efficacy or immunosuppressive treatments [27,28,29]. In exceptional high-risk situations, individual reassessment of risks and benefits may be required when the risk of acquiring the infection is judged to exceed the risk of vaccine-related infection [26,28].

Several guidelines identified vaccines to be considered beyond routine schedule-based vaccination. Influenza vaccination was consistently recommended or emphasized for people with MS, with particular emphasis on patients receiving or scheduled to receive immunosuppressive therapies, those with significant disability, older patients, pregnant women, or other risk factors [24,25,26,27,28,29]. COVID-19 vaccination was addressed in more recent MS guidelines and was recommended for people with MS, with attention to patients at increased risk of severe disease or receiving immunosuppressive therapies [25,27,28]. Pneumococcal vaccination was mainly recommended in relation to immunosuppression, significant disability, or additional risk factors [27,28,29].

Before immunosuppressive treatment, several guidelines recommended checking and updating vaccination status, with specific attention to varicella, measles, mumps, rubella, tetanus, hepatitis B, HPV, herpes zoster, and other infections according to serostatus, age, previous vaccination, planned treatment, and local epidemiology [27,28,29]. These recommendations were generally framed as part of pre-treatment risk assessment, in order to optimize vaccine protection before the start of therapies that could reduce vaccine response or increase the risk of vaccine-related adverse events.

Some guidelines also highlighted that vaccine-induced protection may be reduced under selected disease-modifying therapies, particularly sphingosine-1-phosphate receptor modulators and anti-CD20 therapies, whereas responses appeared preserved or less affected with interferons and, for several vaccines, dimethyl fumarate [27,28,29]. For patients treated with anti-CD20 therapies, more specific timing recommendations advised administering inactivated vaccines, when clinically feasible, at least 3 months after the last infusion and 4–6 weeks before the next infusion to optimize vaccine response [28].

Vaccination during relapse was generally discouraged or delayed until clinical resolution, stabilization, or until the relapse was no longer active [26,28]. More detailed guidance recommended postponing live attenuated vaccines for one month after high-dose corticosteroid pulses; inactivated vaccines should ideally also be delayed for one month, although they may be administered if clinically needed [28]. The SFSEP/France4MS 2024 update similarly recommended adjusting vaccination timing around high-dose methylprednisolone, particularly for live attenuated vaccines [27].

Vaccination of close contacts was recommended or addressed in some MS guidelines, particularly to protect immunocompromised patients [27,28]. Recommended strategies focused mainly on influenza vaccination and on selected vaccines such as MMR and/or varicella according to the susceptibility of household contacts and the protection status of the patient [27,28]. COVID-19 vaccination of close contacts was also addressed [27].

It is worth noting that one MS management guideline [24] referred primarily to the UK Green Book for vaccination recommendations, without providing extensive disease-specific guidance [32]. The Green Book also identifies several neurological and neuromuscular conditions as clinical risk groups for influenza vaccination, including conditions associated with impaired respiratory function, MS, dementia, Parkinson’s disease, motor neuron disease, severe neurological disability, and hereditary or degenerative diseases of the nervous system or muscles. In this handbook, vaccination should be deferred in individuals with evolving neurological conditions until the condition has stabilized or resolved [32].

The cerebral palsy guideline explicitly highlighted the relevance of influenza vaccination for adults with cerebral palsy [23]. For other immunization recommendations, the document referred to the UK Green Book [32]. This confirmed the indication for influenza vaccination in individuals with neurological and neuromuscular conditions, including cerebral palsy, and recommended pneumococcal vaccination for children with respiratory conditions caused by aspiration or neuromuscular disorders associated with aspiration risk. 

The Duchenne muscular dystrophy care considerations included vaccination recommendations within the primary care section [31]. The document recommended administering all non-live-virus vaccinations according to the US Centers for Disease Control and Prevention (CDC) schedule, while aiming to complete live-virus vaccines before the initiation of steroid treatment. Live-virus vaccines were contraindicated in individuals with Duchenne muscular dystrophy receiving high-dose daily corticosteroids. The document placed particular emphasis on prevention of respiratory infections, recommending injectable seasonal influenza vaccination for individuals with Duchenne muscular dystrophy and their close contacts and pneumococcal vaccination.

### 3.5. Results of Syntheses, Reporting Biases, and Certainty of Evidence

Given the nature of the review question and the heterogeneity of the included guideline documents, no quantitative synthesis or meta-analysis was performed. Assessment of reporting biases and certainty of evidence was not applicable, as the review did not pool effect estimates from primary studies.

## 4. Discussion

This review provides an overview of official national and international immunization guidance for people with chronic neurological disorders across selected countries with broadly comparable healthcare and socioeconomic contexts. Overall, vaccination guidance for this population was available, but unevenly distributed across conditions, countries, and document types. 

A first relevant finding concerns the heterogeneity of guideline formats and reporting. Documents labelled as guidelines, consensus statements, handbooks, care considerations, or practical recommendations often differ in methodological transparency and reporting standards. This made identification and comparison of relevant documents challenging, particularly in grey literature sources, where evidence-review methods, recommendation-development processes, and updating procedures were not always clearly described. Clearer terminology, transparent development processes, and more consistent formats could improve both guideline comparability and uptake [33].

National immunization guidelines generally addressed neurological conditions as risk groups within broader vaccine-specific recommendations. This approach is useful for defining vaccine eligibility at population level, but it may be difficult to apply in clinical practice when broad categories such as “neurological” or “neuromuscular” conditions are not further operationalized. Disease-specific neurological guidelines, when available, provided more clinically contextualized recommendations, including treatment timing, contraindications, and special precautions. However, such guidance was available for only a limited number of neurological conditions. This suggests that vaccination is still not consistently integrated as a component of the routine care pathway for many patients with chronic neurological disorders.

Some core messages were consistent across documents. Influenza vaccination was the most frequently recommended or emphasized vaccine in both national immunization guidelines and disease-specific neurological guidelines. COVID-19 vaccination was addressed in more recent documents, particularly for people at increased risk of severe disease or receiving immunosuppressive therapies. Pneumococcal vaccination was less uniformly covered, but was generally recommended in relation to immunosuppression, significant disability, impaired respiratory function, impaired secretion clearance, aspiration risk, or other clinical risk factors. These findings are consistent with the broader rationale for vaccinating vulnerable clinical populations, in whom vaccine-preventable infections may lead to severe complications, hospitalization, or worsening of underlying disease [34,35,36].

MS emerged as the area with the most developed vaccination guidance. This likely reflects the specific challenges posed by disease-modifying therapies, particularly high-efficacy and immunosuppressive treatments, which make vaccine timing, safety, and expected response clinically relevant. Across MS guidelines, routine vaccination was generally supported, but recommendations consistently emphasized the need to assess and update vaccination status early, ideally before treatment initiation. Particular attention was given to live attenuated vaccines, which require careful consideration according to treatment status and degree of immunosuppression, and to therapies that may reduce vaccine-induced protection, such as sphingosine-1-phosphate receptor modulators and anti-CD20 agents. These findings are consistent with broader literature, which supports integrating vaccination planning into therapeutic decision-making when treatments may affect vaccine safety or effectiveness [3,37]. In this context, vaccination becomes part of treatment safety and risk management, supporting early assessment of vaccination status and proactive planning before immunosuppressive therapy is started.

However, the predominance of multiple sclerosis guidelines should not be interpreted as indicating that vaccination is only relevant to this disease. Rather, multiple sclerosis appears to be the condition for which vaccination needs have been most clearly translated into formal neurological guidance. For instance, similar issues may affect other neuroimmunological diseases, including neuromyelitis optica spectrum disorder, myelin oligodendrocyte glycoprotein antibody-associated disease, autoimmune encephalitis, chronic inflammatory demyelinating polyneuropathy, myasthenia gravis, and inflammatory myopathies, which may also require immunomodulatory or immunosuppressive therapies. These treatments can increase infection risk, impair vaccine responses, and affect the safety of live attenuated vaccines. Existing literature therefore supports the need for vaccination planning across neuroimmunological diseases [3], although this need is less often translated into formal disease-specific guidelines. 

During screening, several documents that did not meet the predefined definition of clinical practice guidelines were also identified. These included practical recommendations, expert statements, patient-oriented materials, and operational documents produced by clinicians, scientific groups, or patient organizations. Although these sources were excluded, their presence is informative. They suggest that clinicians and patient organizations often develop pragmatic guidance to address practical questions that are not consistently covered by formal guidelines based on predefined evidence-synthesis and recommendation-development processes. This reinforces the need for future guidelines to combine methodological rigor with operational recommendations that are useful in clinical practice.

From a clinical perspective, the lack of coherent and operational guidance may make vaccination planning more difficult in individuals at increased risk of complications from vaccine-preventable infections [33]. This issue is particularly important for patients receiving, or being evaluated for, disease-modifying or immunosuppressive therapies, for whom the timing and sequencing of immunization may influence both vaccine efficacy and treatment safety [37]. Neurologists, infectious disease specialists, primary care providers, public health services, and vaccination clinics need shared tools that integrate vaccination planning into routine neurological management. Practical instruments such as vaccination checklists, pre-treatment assessment pathways, clinician-facing decision aids, and clear referral criteria could reduce missed opportunities, in line with evidence supporting healthcare professional-targeted interventions to improve vaccine uptake and counselling [38].

Regular updating should also be considered an essential feature of future guidelines, as new vaccines and expanded indications may become relevant for selected neurological populations. Respiratory syncytial virus vaccination provides a useful example. Recent evidence in adults hospitalized with respiratory syncytial virus showed that underlying conditions and multimorbidity were common, and that dementia and neuromuscular conditions were among the conditions considered in risk profiling [39]. 

These findings have relevant public health implications for preventive care in patients with neurological diseases. Clear, accessible, and consistent immunization guidance may help support adherence and counter the effects of vaccine hesitancy and health misinformation [40,41,42]. Greater harmonization across countries, particularly within the same geographical region, could improve consistency in recommendations, while common terminology and structure would enhance accessibility and usability [33,43,44]. Guidance should also be easily available on official government websites for both the public and clinicians involved in vaccine counselling. Communication strategies remain important to improve vaccine adherence [44], especially for patients with chronic neurological conditions, including those receiving immunosuppressive therapies, who may have an increased burden of disease and require tailored vaccination schedules [7,35,36,45]. Based on the heterogeneity observed in this review, harmonized international frameworks and minimum standards for immunization recommendations could support both patients and clinicians and may help reduce inequities across healthcare settings.

From a clinical perspective, the findings support the integration of vaccination assessment into routine neurological care. Vaccination status should be reviewed early, particularly before initiation of immunosuppressive or immunomodulatory therapies, and clinicians should consider respiratory, functional, treatment-related, and care-related vulnerability when identifying patients who may need targeted immunization advice. Practical tools such as vaccination checklists, pre-treatment assessment pathways, referral criteria, and coordination between neurology, infectious disease, primary care, and vaccination services may help reduce missed opportunities and improve the implementation of existing recommendations.

Several limitations should be acknowledged. Restricting the review to countries with a Human Development Index greater than 0.8 helped delimit the scope of the review and reduce broad socioeconomic heterogeneity. However, HDI does not fully reflect the complexity of national healthcare and immunization systems. Therefore, this criterion may have limited generalizability and introduced selection bias toward higher-resource settings. The search was restricted to languages known to the review team. Although these languages covered several major European and international sources, relevant national guidelines published in other languages may have been missed, introducing potential language bias. Grey literature searches are inherently affected by website structure, indexing, accessibility, and changes over time, and some documents may not have been digitally available. The bibliographic search was limited to three databases, PubMed/MEDLINE, Embase, and Scopus, and did not include all potentially relevant bibliographic sources. However, this limitation was partly mitigated by the systematic grey literature search, reference list screening, and checks of issuing organizations’ websites for updated or related official documents. Inter-reviewer agreement coefficients, such as Cohen’s kappa, were not calculated for the title/abstract or full-text screening stages. Finally, the review included only documents meeting the predefined definition of clinical practice guidelines or equivalent structured guidance; therefore, practical recommendations, expert opinion documents, and patient-oriented materials were excluded even when they contained clinically relevant advice. These limitations are partly balanced by several strengths. The review used a broad search strategy covering both bibliographic databases and grey literature, thereby increasing the likelihood of identifying relevant guidance documents across different publication channels. The country-by-country grey literature search was particularly important, as it allowed identification of official documents that are not necessarily indexed in scientific databases. To our knowledge, this is the first systematic review specifically focused on official guidance for preventive immunization in people with chronic neurological disorders.

## 5. Conclusions

Official vaccination guidance for people with chronic neurological disorders remains limited and unevenly distributed. While multiple sclerosis has the most developed recommendations, vaccination should be considered a broader component of neurological care, relevant not only to treatment-related immunosuppression but also to respiratory, functional, and care-related vulnerability. Future guidelines should move from generic risk-group definitions to clear, evidence-based and operational recommendations that support timely vaccination assessment, treatment-sensitive planning, multidisciplinary coordination, and regular updating as new vaccines and indications become available.

## Figures and Tables

**Table 1:** Overview of the included guidelines by country or region, issuing body, year, and recommendation focus.

Country/ Region	Issuing Body	Publication Year ^a^	Vaccine ^b^	Disease ^c^
Argentina [29]	Argentine Neurological Society (SNA)	2021	X	X (MS)
Australia [18]	Department of Health and Aged Care, Australian Government	2024	X	
Canada [19]	Public Health Agency of Canada	2024	X	
Europe [28]	ECTRIMS/EAN	2023	X	X (MS)
France [27]	France4MS, SFSEP	2024	X	X (MS)
Italy [25]	Italian Society of Neurology (SIN)	2022		X (MS)
New Zealand [20]	Health New Zealand Te Whatu Ora	2024	X	
Singapore [21]	Society of Infectious Disease	2019	X	
UK [24]	National Institute for Health and Care Excellence (NICE)	2022		X (MS)
UK [23]	National Institute for Health and Care Excellence (NICE)	2019		X (CP)
USA [31]	DMD Care Considerations Working Group (with CDC support)	2018		X (DMD)
USA [26]	American Academy of Neurology (AAN)	2019	X	X (MS)

^a^ For resources accessible only as online websites, the year of access is reported. ^b^ Flagged if the document addresses vaccines and includes a section dedicated to patients with chronic neurological diseases. ^c^ Flagged if the document addresses chronic neurological diseases and includes a section dedicated to vaccines. Abbreviations: CDC Centers for Disease Control and Prevention; CP Cerebral palsy; DMD Duchenne muscular dystrophy; ECTRIMS/EAN European Committee for Treatment and Research in Multiple Sclerosis/European Academy of Neurology; France4MS, French Group for Recommendations in Multiple Sclerosis; MS Multiple Sclerosis; SFSEP, French Multiple Sclerosis Society; UK United Kingdom; USA United States of America.

**Table 2:** Summary of recommendations from national immunization guidelines including neurological conditions.

Area	Summary of Recommendations	Guidelines
Routine immunisation	Routine vaccination was generally considered appropriate in individuals with stable neurological conditions, provided that no other vaccine-specific contraindications were present.	[18,19,20,21]
Pertussis-containing vaccines	Variable recommendations: postponement in progressive/uncontrolled disorders, individual assessment in unstable conditions, and contraindication after previous vaccine-associated encephalopathy.	[18,20,21]
Influenza vaccination	Recommended for people with neuromuscular or central nervous system conditions	[18,19,20,21]
COVID-19 vaccination	Recommended for a broad range of neurological condition	[20]
Pneumococcal vaccination	Addressed in two guidelines; one provided detailed risk-based indications for neurological groups, including cerebrospinal fluid leak and impaired clearance of oral secretions.	[19,20]
Immunosuppression and live vaccines	Live vaccines contraindicated in immunocompromised individuals; one guideline explicitly linked this to neurological conditions requiring immunosuppressive therapies.	[19]

**Table 3:** Summary of recommendations from multiple sclerosis guidelines.

Area	Summary of Recommendations	Guidelines
Routine vaccination	Generally considered appropriate, unless specific contraindications were present.	[26,27,28,29]
Timing of vaccination	Vaccination status should be assessed and updated early in the disease course and before initiating disease-modifying, immunosuppressive, immunomodulatory, or high-efficacy therapies.	[25,26,27,28,29]
Live attenuated vaccines	Generally contraindicated during treatment with immunosuppressive or disease-modifying therapies and after recent discontinuation. In exceptional high-risk situations, individual reassessment of risks and benefits may be required.	[26,27,28,29]
Influenza vaccination	Specifically recommended for patients with multiple sclerosis.	[24,26,27,28,29]
COVID-19 vaccination	Specifically recommended for patients with multiple sclerosis.	[25,27,28]
Pneumococcal vaccination	Recommended mainly in relation to immunosuppression, significant disability, or risk factors.	[27,28,29]
Other vaccinations before immunosuppressive treatment	Check and update vaccination status before treatment; specific attention to varicella/VZV, measles, mumps, rubella, tetanus, hepatitis B, HPV, herpes zoster and other infections according to serostatus, age, previous vaccination, planned treatment and local epidemiology.	[27,28,29]
Close contacts	Recommended vaccination of close contacts against influenza, MMR and/or varicella [27,28], and COVID-19 [27], particularly for immunocompromised patients	[27,28]

## Data Availability

No new data were created or analyzed in this study. Data sharing is not applicable to this article.

## Supplementary Materials

The following supporting information can be downloaded at: https://www.mdpi.com/article/10.3390/vaccines14090777/s1, Supplementary Section S1: Chronic neurological disorders; Supplementary Section S2: Included countries; Supplementary Section S3: Search strategy; Supplementary Section S4: Quality appraisal.

## Abbreviations

The following abbreviations are used in this manuscript:

AAN	American Academy of Neurology
AGREE II	Appraisal of Guidelines for Research and Evaluation II
CDC	Centers for Disease Control and Prevention
COVID-19	Coronavirus disease 2019
CP	Cerebral palsy
DMD	Duchenne muscular dystrophy
ECTRIMS/EAN	European Committee for Treatment and Research in Multiple Sclerosis/European Academy of Neurology
France4MS	French Group for Recommendations in Multiple Sclerosis
HDI	Human Development Index
HPV	Human papillomavirus
ICD-11	International Classification of Diseases, 11th Revision
IOM	Institute of Medicine
MMR	Measles, mumps, and rubella
MS	Multiple sclerosis
NICE	National Institute for Health and Care Excellence
PICo	Population, Phenomenon of Interest, Context
PRISMA	Preferred Reporting Items for Systematic Reviews and Meta-Analyses
PROSPERO	International Prospective Register of Systematic Reviews
RSV	Respiratory syncytial virus
SFSEP	Société Francophone de la Sclérose en Plaques
SNA	Argentine Neurological Society
UK	United Kingdom
USA	United States of America
