# Supplementary material for: Guidelines on Immunization of People with Chronic Neurological Disorders: A Systematic Review
Source: Vaccines (Basel). 2026 Sep 5;14(9):777. doi: 10.3390/vaccines14090777 (PMC13611198; doi:10.3390/vaccines14090777)
Supplement: Supplementary file 1 [file vaccines-14-00777-s001.zip › vaccines-4501568-supplementary.pdf]

SUPPLEMENTARY MATERIAL

Guidelines on immunization of people with chronic neurological disorders: a systematic review

Giuseppina Lo Moro[1], Andreea Mihaela Butnaru[1], Carola Toffanin[1*], Anastasia Cataldo[1*], Simona Mazzeo[1], Manuela Martella[1], Giacomo Scaioli[1], Fabrizio Bert[1†], and Roberta Siliquini[1,2†]

[1] Department of Public Health and Pediatric Sciences, University of Turin, 10126, Torino, Italy;
[2] A.O.U. City of Health and Science of Torino, 10126, Torino, Italy.
* Correspondence
†These authors contributed equally to this work and share last authorship.

Table of contents

SECTION 2
S1. Chronic neurological disorders 2
S2. Included countries 6
S3. Search strategy 8
S4. Quality appraisal 12

Academic Editor: Nobuhiko Okabe

Received: 30 July 2026
Revised: 25 August 2026
Accepted: 3 September 2026
Published: 5 September 2026

Copyright: © 2026 by the authors. Submitted for possible open access publication under the terms and conditions of the Creative Commons Attribution (CC BY) license.

SECTION

S1. Chronic neurological disorders

GROUP	DISEASES
**MOVEMENT DISORDERS**	○ Parkinsonism (Parkinson)<br>○ Choreiform disorders (Chorea)<br>○ Dystonic disorders (Dystonia)<br>○ Ataxic disorders (Ataxia)<br>○ Disorders associated with tremor<br>○ Tic disorders<br>○ Myoclonic disorders (Myoclonus)
**DISORDERS WITH NEUROCOGNITIVE IMPAIRMENT AS A MAJOR FEATURE**	○ Alzheimer disease<br>○ Progressive focal atrophies (Atrophy)<br>○ Lewy body disease<br>○ Frontotemporal lobar degeneration
**MULTIPLE SCLEROSIS OR OTHER WHITE MATTER DISORDERS**	○ Multiple sclerosis<br>○ Isolated demyelinating syndromes of the central nervous system<br>● Transverse myelitis<br>● Optic neuritis<br>○ Acute disseminated encephalomyelitis (ADEM)<br>○ Neuromyelitis optica<br>○ Leukodystrophies<br>● Pelizaeus-Merzbacher disease<br>● Adrenoleukodystrophy<br>● Alexander disease<br>● Krabbe disease<br>● Metachromatic leukodystrophy<br>○ Secondary white matter disorders<br>○ Central demyelination of corpus callosum
**EPILEPSY OR SEIZURES**	○ Epilepsy<br>○ Status epilepticus
**CEREBROVASCULAR DISEASES**	○ Intracranial hemorrhage<br>○ Cerebral ischaemia<br>○ Stroke not known if ischaemic or haemorrhagic<br>○ Cerebrovascular disease with no acute cerebral symptom<br>○ Cerebrovascular abnormalities<br>○ Hypoxic-ischaemic encephalopathy

**SPINAL CORD DISORDERS EXCLUDING TRAUMA**	○ Cauda equina syndrome<br>○ Myelitis<br>○ Myelopathy<br>○ Non-compressive vascular myelopathies<br>○ Degenerative myelopathic disorders
**MOTOR NEURON DISEASES OR RELATED DISORDERS**	○ Motor neuron disease<br>■ Amyotrophic lateral sclerosis<br>■ Progressive bulbar palsy<br>■ Progressive muscular atrophy<br>■ Primary lateral sclerosis<br>■ Monomelic amyotrophy<br>■ Madras type motor neuron disease<br>○ Spinal muscular atrophy
**DISORDERS OF NERVE ROOT, PLEXUS OR PERIPHERAL NERVES**	○ Disorders of cranial nerves<br>○ Nerve root or plexus disorders<br>○ Polyneuropathy<br>○ Mononeuropathy<br>○ Hereditary neuropathy
**DISEASES OF NEUROMUSCULAR JUNCTION OR MUSCLE**	○ Myasthenia gravis<br>○ Primary disorders of muscles<br>■ Muscular dystrophy<br>■ Myotonic disorders<br>■ Congenital myopathies<br>■ Mitochondrial myopathies<br>■ Periodic paralyses or disorders of muscle membrane excitability<br>■ Distal myopathies<br>■ Myofibrillar myopathy<br>■ Ocular myopathy<br>○ Secondary myopathies
**CEREBRAL PALSY**	○ Spastic cerebral palsy<br>○ Dyskinetic cerebral palsy<br>○ Ataxic cerebral palsy<br>○ Worster-Drought syndrome
**DISORDERS OF CEREBROSPINAL FLUID PRESSURE OR FLOW**	○ Increased intracranial pressure<br>○ Intracranial hypotension<br>○ Cerebrospinal fluid rhinorrhoea<br>○ Cerebrospinal fluid otorrhoea<br>○ Hydrocephalus<br>○ Cerebrospinal fluid fistula<br>○ Syringomyelia or syringobulbia<br>○ Intracranial arachnoid cyst<br>○ Porencephalic cyst

**DISORDERS OF AUTONOMIC NERVOUS SYSTEM**	○ Congenital malformations of the autonomic nervous system<br>○ Inherited autonomic nervous system disorders<br>○ Autoimmune disorders involving the autonomic nervous system<br>○ Autonomic nervous system disorder<br>○ Autonomic neuropathies<br>○ Disorders of orthostatic tolerance<br>○ Focal or segmental autonomic disorders<br>○ Disorders affecting autonomic synaptic neurotransmission<br>○ Autonomic dysreflexia<br>○ Hypoglycaemia unawareness<br>○ Paroxysmal autonomic disorders<br>○ Hypohidrosis
**NEOPLASMS OF THE NERVOUS SYSTEM**	○ Gliomas, glioneuronal tumors, and neuronal tumors<br>■ Astrocytoma<br>■ Oligodendroglioma<br>■ Glioblastoma<br>■ Neuroepithelial tumor<br>■ Astroblastoma<br>■ Ganglioglioma<br>■ Glioneuronal tumor<br>■ Gangliocytoma<br>■ Neuronal tumor<br>■ Neurocytoma<br>■ Ependymoma<br>○ Choroid plexus tumors<br>■ Choroid plexus papilloma<br>■ Choroid plexus carcinoma<br>○ Embryonal tumors<br>■ Medulloblastoma<br>■ Teratoid/rhabdoid tumor<br>○ Pineal tumors<br>■ Pineocytoma<br>■ Pineal parenchymal tumor of intermediate differentiation<br>■ Pineoblastoma<br>■ Papillary tumor<br>■ Desmoplastic myxoid tumor<br>○ Cranial and paraspinal nerve tumors<br>■ Schwannoma<br>■ Neurofibroma<br>■ Perineurioma

	■ Nerve sheath tumor<br>■ Paraganglioma<br>○ Meningioma<br>○ Mesenchymal tumors<br>■ Fibroblastic and myofibroblastic tumors<br>● Solitary fibrous tumor<br>■ Vascular tumors<br>● Hemangiomas and vascular malformations<br>● Hemangioblastoma<br>■ Skeletal muscle tumors<br>● Rhabdomyosarcoma<br>■ Ewing sarcoma<br>■ Chondrosarcoma<br>○ Melanocytic tumors<br>○ Hematolymphoid tumors<br>■ Lymphomas<br>■ Histiocytic tumors<br>○ Germ cell tumors<br>■ Teratoma<br>■ Germinoma<br>■ Embryonal carcinoma<br>■ Yolk sac tumor<br>■ Choriocarcinoma<br>○ Tumors of the sellar region<br>■ Craniopharyngioma<br>■ Pituicytoma<br>■ Pituitary adenoma/blastoma<br>○ Metastases
**STRUCTURAL DEVELOPMENTAL ANOMALIES OF THE NERVOUS SYSTEM**	○ Anencephaly or similar anomalies<br>○ Cephalocele<br>○ Spina bifida<br>○ Arnold-Chiari malformation type II<br>○ Congenital hydrocephalus<br>○ Cerebral structural developmental anomalies<br>○ Cerebellar structural developmental anomalies<br>○ Structural developmental anomalies of the neurenteric canal, spinal cord or vertebral column

S2. Included countries

1) Andorra
2) Argentina
3) Australia
4) Austria
5) Bahamas
6) Bahrain
7) Belarus
8) Belgium
9) Brunei
10) Canada
11) Chile
12) Costa Rica
13) Croatia
14) Cyprus
15) Czech
16) Republic
17) Denmark
18) Estonia
19) Finland
20) France
21) Georgia
22) Germany
23) Greece
24) Hong Kong
25) Hungary
26) Iceland
27) Ireland
28) Israel
29) Italy
30) Japan
31) Kazakhstan
32) Kuwait
33) Latvia
34) Liechtenstein
35) Lithuania
36) Luxembourg
37) Malaysia
38) Malta
39) Mauritius
40) Montenegro
41) Netherlands
42) New Zealand
43) Norway
44) Oman
45) Panama
46) Poland
47) Portugal
48) Qatar
49) Romania

50) Russia
51) San Marino
52) Saudi Arabia
53) Serbia
54) Singapore
55) Slovakia
56) Slovenia
57) South Korea
58) Spain
59) Sweden
60) Switzerland
61) Thailand
62) Trinidad and Tobago
63) Turkey
64) United Arab Emirates
65) United Kingdom
66) United State
67) Uruguay

S3. Search strategy

1) PubMed:

(("Guideline" [Publication Type] OR "Guidelines as Topic"[Mesh] OR "Health Planning Guidelines"[Mesh] OR guidelin*)

AND

("Nervous System Diseases"[Mesh] OR "Nervous System Disease*"[tiab] OR “neurologic* disorder*”[tiab] OR “neurologic* disease*”[tiab] OR “nervous system disorder*”[tiab] OR “nerv*”[tiab] OR “neurologic*”[tiab] OR “Parkinson*” [tiab] OR “Chore*”[tiab] OR “Dystoni*”[tiab] OR “Ataxi*”[tiab] OR “tremor*”[tiab] OR “tic”[tiab] OR “Myoclon*”[tiab] OR “Alzheimer”[tiab] OR “Progressive focal atroph*”[tiab] OR “Lewy body disease”[tiab] OR “Frontotemporal lobar degeneration”[tiab] OR “Multiple sclerosis”[tiab] OR “Transverse myelitis”[tiab] OR “Optic neuritis”[tiab] OR “Acute disseminated encephalomyelitis”[tiab] OR “Neuromyelitis optica”[tiab] OR “Leukodystroph*”[tiab] OR “Pelizaeus-Merzbacher disease”[tiab] OR “Adrenoleukodystrophy”[tiab] OR “Alexander disease”[tiab] OR “Krabbe disease”[tiab] OR “White matter disorder*”[tiab] OR “corpus callosum demyelination”[tiab] OR “Epilep*”[tiab] OR “Seizur*”[tiab] OR “Status epilepticus”[tiab] OR “Intracranial hemorrhag*”[tiab] OR “Cerebral ischaemi*”[tiab] OR “Stroke”[tiab] OR “Cerebrovascular abnormalit*”[tiab] OR “Hypoxic-ischaemic encephalopathy”[tiab] OR “Cauda equina syndrome”[tiab] OR “Myelitis*”[tiab] OR “Myelopath*”[tiab] OR “motor neuron disease*”[tiab] OR “Amyotrophic lateral sclerosis”[tiab] OR “Progressive bulbar palsy”[tiab] OR “Progressive muscular atrophy”[tiab] OR “Primary lateral sclerosis”[tiab] OR “Monomelic amyotrophy”[tiab] OR “Madras type motor neuron disease”[tiab] OR “spinal muscular atrophy”[tiab] OR “Cranial nerv* disorder*”[tiab] OR “nerve root disorder*”[tiab] OR “plexus disorder*”[tiab] OR “Neuropath*”[tiab] OR “Myasthenia gravis”[tiab] OR “Muscular dystroph*”[tiab] OR “Myotonic disorder*”[tiab] OR “Myopath*”[tiab] OR “Periodic paralys*”[tiab] OR “Muscle membrane excitability disorder*”[tiab] OR “Cerebral palsy”[tiab] OR “Worster Drought syndrome”[Supplementary Concept] OR “Worster Drought syndrome”[tiab] OR “Increased intracranial pressure”[tiab] OR “Intracranial hypertension”[tiab] OR “Intracranial hypotension”[tiab] OR “Cerebrospinal fluid rhinorrhoea”[tiab] OR “Cerebrospinal fluid otorrhoea”[tiab] OR “Hydrocephalus”[tiab] OR “Cerebrospinal fluid fistula”[tiab] OR “syringomyelia”[tiab] OR “Syringobulbia”[tiab] OR “intracranial arachnoid cyst”[tiab] OR “Porencephalic cyst”[tiab] OR “autonomic malformation*”[tiab] OR “Autonomic disorder*”[tiab] OR “Autonomic neuropath*”[tiab] OR “Autonomic dysreflexia”[tiab] OR “Hypoglycaemia unawareness”[tiab] OR “Paroxysmal autonomic disorder*”[tiab] OR “Hypohidrosis”[tiab] OR “orthostatic tolerance disorder*”[tiab] OR "Glioma"[Mesh] OR "Neoplasms, Neuroepithelial"[Mesh] OR "Teratoma"[Mesh] OR "Neoplasms, Nerve Tissue"[Mesh] OR "Hemangioma"[Mesh] OR "Myosarcoma"[Mesh] OR "Sarcoma, Ewing"[Mesh] OR "Chondrosarcoma"[Mesh] OR "Neoplasms, Germ Cell and Embryonal"[Mesh] OR "Craniopharyngioma"[Mesh] OR “Nervous system neoplasm*”[tiab] OR “Nervous system tumor*”[tiab] OR “Nervous system cancer*”[tiab] OR “Glioma”[tiab] OR “Glioneuronal tumor*”[tiab] OR “Neuronal tumor*”[tiab] OR “Astrocytoma”[tiab] OR “Oligodendroglioma”[tiab] OR “Glioblastoma”[tiab] OR “Neuroepithelial tumor”[tiab] OR “Astroblastoma”[tiab] OR “Ganglioglioma”[tiab] OR “Gangliocytoma”[tiab] OR “Neurocytoma”[tiab] OR “Ependymoma”[tiab] OR “Choroid plexus tumor*”[tiab] OR “Choroid plexus papilloma”[tiab] OR “Choroid plexus carcinoma”[tiab] OR “Medulloblastoma”[tiab] OR “Teratoid tumor*”[tiab] OR “Rhabdoid tumor*”[tiab] OR “Pineal tumor*”[tiab] OR “Pineocytoma”[tiab] OR “Pineal parenchymal tumor*”[tiab] OR “Pineoblastoma”[tiab] OR “Papillary tumor*”[tiab] OR “Desmoplastic myxoid tumor*”[tiab] OR “Schwannoma”[tiab] OR “Neurofibroma”[tiab] OR “Perineurioma”[tiab] OR “Nerve sheath tumor”[tiab] OR “Paraganglioma”[tiab] OR “Meningioma”[tiab] OR “Mesenchymal tumor*”[tiab] OR “Fibroblastic tumor*”[tiab] OR “myofibroblastic tumor*”[tiab] OR “Solitary fibrous tumor

Vascular tumor*”[tiab] OR “Hemangioma”[tiab] OR “vascular malformation*”[tiab] OR “Hemangioblastoma”[tiab] OR “skeletal muscle tumor*”[tiab] OR “Rhabdomyosarcoma”[tiab] OR “Ewing Sarcoma”[tiab] OR “Chondrosarcoma”[tiab] OR “Melanocytic tumor*”[tiab] OR “Hematolymphoid

tumor*”[tiab] OR “Lymphoma*”[tiab] OR “Histiocytic tumor*”[tiab] OR “Germ cell tumor*”[tiab] OR “Teratoma”[tiab] OR “Germinoma”[tiab] OR “Embryonal carcinoma”[tiab] OR “Yolk sac tumor”[tiab] OR “Choriocarcinoma”[tiab] OR “sellar region tumor*”[tiab] OR “Craniopharyngioma”[tiab] OR “Pituicytoma”[tiab] OR “Pituitary adenoma”[tiab] OR “Pituitary blastoma”[tiab] OR “Metastase*”[tiab] OR “Anencephaly"[tiab] OR “Cephalocele”[tiab] OR “Spina bifida”[tiab] OR “Arnold-Chiari malformation*”[tiab] OR “Congenital hydrocephalus”[tiab] OR “Cerebral structural developmental anomal*”[tiab] OR “Cerebellar structural developmental anomal*”[tiab] OR “spinal cord developmental anomal*”[tiab] OR “vertebral column developmental anomal*”[tiab])

AND

(Vaccin*[tiab] OR immuniz*[tiab] OR immunis*[tiab] OR “Vaccines”[Mesh] OR “Immunization”[Mesh] OR “Vaccination”[Mesh]))

2) Embase:

('practice guideline'/exp OR ‘guideline*’) AND ('neurologic* disease*'/exp OR ‘neurologic* disorder*’:ab,ti OR ‘neurologic* disease*’:ab,ti OR ‘nervous system disorder*’:ab,ti OR 'autonomic dysfunction'/exp OR 'motor dysfunction'/exp OR 'cerebrovascular disease'/exp OR ‘Monomelic amyotrophy’:ab,ti OR ‘Madras motor neuron disease’:ab,ti OR 'myotonia'/exp OR ‘Parkinson*’:ab,ti OR ‘Chore*’:ab,ti OR ‘Dystoni*’:ab,ti OR ‘Ataxi*’:ab,ti OR ‘tremor*’:ab,ti OR ‘tic’:ab,ti OR ‘Myoclon*’:ab,ti OR ‘Alzheimer’:ab,ti OR ‘Progressive focal atroph*’:ab,ti OR ‘Lewy body disease’:ab,ti OR ‘Frontotemporal lobar degeneration’:ab,ti OR ‘multiple sclerosis’:ab,ti OR ‘Transverse myelitis’:ab,ti OR ‘Optic neuritis’:ab,ti OR ‘Acute disseminated encephalomyelitis’:ab,ti OR ‘Neuromyelitis optica’:ab,ti OR ‘Leukodystroph*’:ab,ti OR ‘Pelizaeus-Merzbacher disease’:ab,ti OR ‘Adrenoleukodystrophy’:ab,ti OR ‘Alexander disease’:ab,ti OR ‘Krabbe disease’:ab,ti OR ‘ White matter disorder*’:ab,ti OR ‘Corpus callosum demyelination':ab,ti OR ‘epilep*’:ab,ti OR ‘seizur*’:ab,ti OR ‘status epilepticus’:ab,ti OR ‘Intracranial hemorrhag*’:ab,ti OR ‘Cerebral ischaemi*’:ab,ti OR ‘stroke’:ab,ti OR ‘Cerebrovascular disease*’:ab,ti OR ‘Cerebrovascular abnormalit*’:ab,ti OR ‘Hypoxic-ischaemic encephalopathy’:ab,ti OR ‘Cauda equina syndrome’:ab,ti OR ‘Myelitis*’:ab,ti OR ‘Myelopath*’:ab,ti OR ‘Motor neuron disease*’:ab,ti OR ‘Amyotrophic lateral sclerosis’:ab,ti OR ‘Progressive bulbar palsy’:ab,ti OR ‘Progressive muscular atrophy’:ab,ti OR ‘Primary lateral sclerosis’:ab,ti OR ‘Monomelic amyotrophy’:ab,ti OR ‘Spinal muscular atrophy’:ab,ti OR ‘Cranial nerv* disorder*’:ab,ti OR ‘Nerve root disorder*’:ab,ti OR ‘plexus disorder*’:ab,ti OR ‘neuropath*’:ab,ti OR ‘Myasthenia gravis’:ab,ti OR ‘Muscular dystroph*’:ab,ti OR ‘Myotonic disorder*’:ab,ti OR ‘myopath*’:ab,ti OR ‘periodic paralys*’:ab,ti OR ‘muscle membrane excitability disorder*’:ab,ti OR ‘cerebral palsy’:ab,ti OR ‘Worster-Drought syndrome’:ab,ti OR ‘Increased intracranial pressure’:ab,ti OR ‘intracranial hypertension’:ab,ti OR ‘Intracranial hypotension’:ab,ti OR ‘Cerebrospinal fluid rhinorrhoea’:ab,ti OR ‘Cerebrospinal fluid otorrhoea’:ab,ti OR ‘Hydrocephalus’:ab,ti OR ‘Cerebrospinal fluid fistula’:ab,ti OR ‘Syringomyelia’:ab,ti OR ‘syringobulbia’:ab,ti OR ‘Intracranial arachnoid cyst’:ab,ti OR ‘Porencephalic cyst’:ab,ti OR ‘autonomic malformation*’:ab,ti OR ‘autonomic disorder*’:ab,ti OR ‘Autonomic neuropath*’:ab,ti OR ‘Autonomic dysreflexia’:ab,ti OR ‘Hypoglycaemia unawareness’:ab,ti OR ‘Paroxysmal autonomic disorder*’:ab,ti OR ‘Hypohidrosis’:ab,ti OR ‘orthostatic tolerance disorder*’:ab,ti OR ‘nervous system neoplasm*’:ab,ti OR ‘neervous system tumor*’:ab,ti OR ‘Nervous system cancer*’:ab,ti OR ‘glioma’:ab,ti OR ‘glioneuronal tumor*’:ab,ti OR ‘Neuronal tumor*’:ab,ti OR ‘Astrocytoma’:ab,ti OR ‘Oligodendroglioma’:ab,ti OR ‘Glioblastoma’:ab,ti OR ‘Neuroepithelial tumor’:ab,ti OR ‘Astroblastoma’:ab,ti OR ‘Ganglioglioma’:ab,ti OR ‘Gangliocytoma’:ab,ti OR ‘Neurocytoma’:ab,ti OR ‘Ependymoma’:ab,ti OR ‘Choroid plexus tumor*’:ab,ti OR ‘Choroid plexus papilloma’:ab,ti OR ‘Choroid plexus carcinoma’:ab,ti OR ‘Medulloblastoma’:ab,ti OR ‘Teratoid tumor’:ab,ti OR ‘rhabdoid tumor’:ab,ti OR ‘Pineocytoma’:ab,ti OR ‘Pineal tumor’:ab,ti OR ‘Pineoblastoma’:ab,ti OR ‘Papillary tumor’:ab,ti OR ‘Desmoplastic myxoid tumor’:ab,ti OR ‘Schwannoma’:ab,ti OR ‘Neurofibroma’:ab,ti OR ‘Perineurioma’:ab,ti OR ‘Nerve sheath tumor’:ab,ti OR ‘Paraganglioma’:ab,ti OR ‘Meningioma’:ab,ti OR ‘Mesenchymal tumor*’:ab,ti OR ‘fibroblastic tumor*’:ab,ti OR ‘myofibroblastic tumor’:ab,ti OR ‘Solitary fibrous

tumor’:ab,ti OR ‘vascular tumor*’:ab,ti OR ‘Hemangioma’:ab,ti OR ‘vascular malformation*’:ab,ti OR ‘Hemangioblastoma’:ab,ti OR ‘Skeletal muscle tumor*’ OR ‘Rhabdomyosarcoma’:ab,ti OR ‘Ewing sarcoma’:ab,ti OR ‘Chondrosarcoma’:ab,ti OR ‘Melanocytic tumor’:ab,ti OR ‘Hematolymphoid tumor*’:ab,ti OR ‘Lymphoma’:ab,ti OR ‘Histiocytic tumor’:ab,ti OR ‘Germ cell tumor*’:ab,ti OR ‘Teratoma’:ab,ti OR ‘Germinoma’:ab,ti OR ‘Embryonal carcinoma’:ab,ti OR ‘Yolk sac tumor’:ab,ti OR ‘Choriocarcinoma’:ab,ti OR ‘sellar region tumor*’:ab,ti OR ‘Craniopharyngioma’:ab,ti OR ‘Pituicytoma’:ab,ti OR ‘Pituitary adenoma’:ab,ti OR ‘Pituitary blastoma’:ab,ti OR ‘metastase*’:ab,ti OR ‘Anencephaly’:ab,ti OR ‘Cephalocele’:ab,ti OR ‘Spina bifida’:ab,ti OR ‘Arnold-Chiari malformation’:ab,ti OR ‘Congenital hydrocephalus’:ab,ti OR ‘Cerebral structural developmental anomal*’:ab,ti OR ‘Cerebellar structural developmental anomal*’:ab,ti OR ‘spinal cord developmental anomal*’:ab,ti OR ‘vertebral column developmental anomal*’:ab,ti)

AND (Vaccin*:ab,ti OR immuniz*:ab,ti OR immunis*:ab,ti OR 'vaccine'/exp OR 'immunization'/exp)

3) Scopus:

(Guidelin* OR INDEXTERMS(Guidelin*))

AND

(TITLE-ABS(Nervous System Disease*) OR TITLE-ABS(Neurologic* disorder*) OR TITLE-ABS(neurologic* disease*) OR TITLE-ABS(nervous system disorder*) OR TITLE-ABS(neurologic*) OR TITLE-ABS(nerv*) OR TITLE-ABS(Parkinson*) OR TITLE-ABS(Chore*) OR TITLE-ABS(Dystoni*) OR TITLE-ABS(Ataxi*) OR TITLE-ABS(tremor*) OR TITLE-ABS(tic) OR TITLE-ABS(Myoclon*) OR TITLE-ABS(Alzheimer) OR TITLE-ABS(Progressive focal atroph*) OR TITLE-ABS(Lewy body disease) OR TITLE-ABS(Frontotemporal lobar degeneration) OR TITLE-ABS(Multiple sclerosis) OR TITLE-ABS(Transverse myelitis) OR TITLE-ABS(Optic neuritis) OR TITLE-ABS(Acute disseminated encephalomyelitis) OR TITLE-ABS(Neuromyelitis optica) OR TITLE-ABS(Leukodystroph*) OR TITLE-ABS(Pelizaeus-Merzbacher disease) OR TITLE-ABS(Adrenoleukodystrophy) OR TITLE-ABS(Alexander disease) OR TITLE-ABS(Krabbe disease) OR TITLE-ABS(White matter disorder*) OR TITLE-ABS(Corpus callosum demyelination) OR TITLE-ABS(Epilep*) OR TITLE-ABS(seizur*) OR TITLE-ABS(Status epilepticus) OR TITLE-ABS(Intracranial hemorrhag*) OR TITLE-ABS(Cerebral ischaemi*) OR TITLE-ABS(stroke) OR TITLE-ABS(Cerebrovascular disease*) OR TITLE-ABS(Cerebrovascular abnormalit*) OR TITLE-ABS(Hypoxic-ischaemic encephalopathy) OR TITLE-ABS(Cauda equina syndrome) OR TITLE-ABS(Myelitis*) OR TITLE-ABS(Myelopath*) OR TITLE-ABS(Motor neuron disease*) OR TITLE-ABS(Amyotrophic lateral sclerosis) OR TITLE-ABS(Progressive bulbar palsy) OR TITLE-ABS(Progressive muscular atrophy) OR TITLE-ABS(Primary lateral sclerosis) OR TITLE-ABS(Monomelic amyotrophy) OR TITLE-ABS(Madras type motor neuron disease) OR TITLE-ABS(Spinal muscular atrophy) OR TITLE-ABS(Cranial nerv* disorder*) OR TITLE-ABS(Nerve root disorder*) OR TITLE-ABS(Plexus disorder*) OR TITLE-ABS(Neuropath*) OR TITLE-ABS(Myasthenia gravis) OR TITLE-ABS(Muscular dystroph*) OR TITLE-ABS(Myotonic disorder*) OR TITLE-ABS(Myopath*) OR TITLE-ABS(Periodic paralys*) OR TITLE-ABS(Muscle membrane excitability disorder*) OR TITLE-ABS(Cerebral palsy) OR TITLE-ABS(Worster-Drought syndrome) OR TITLE-ABS(Increased intracranial pressure) OR TITLE-ABS(Intracranial hypertension) OR TITLE-ABS(Intracranial hypotension) OR TITLE-ABS(Cerebrospinal fluid rhinorrhoea) OR TITLE-ABS(Cerebrospinal fluid otorrhoea) OR TITLE-ABS(Hydrocephalus) OR TITLE-ABS(Cerebrospinal fluid fistula) OR TITLE-ABS(Syringomyelia) OR TITLE-ABS(syringobulbia) OR TITLE-ABS(Intracranial arachnoid cyst) OR TITLE-ABS(Porencephalic cyst) OR TITLE-ABS(Autonomic malformation*) OR TITLE-ABS(Autonomic disorder*) OR TITLE-ABS(Autonomic neuropath*) OR TITLE-ABS(Autonomic dysreflexia) OR TITLE-ABS(Hypoglycaemia unawareness) OR TITLE-ABS(Paroxysmal autonomic disorder*) OR TITLE-ABS(Hypohidrosis orthostatic tolerance disorder*) OR TITLE-ABS(Nervous system neoplasm*) OR TITLE-ABS(Nervous system tumor*) OR TITLE-ABS(Nervous system cancer*) OR TITLE-ABS(Glioma)

OR TITLE-ABS(Glioneuronal tumor*) OR TITLE-ABS(Neuronal tumor*) OR TITLE-ABS(Astrocytoma) OR TITLE-ABS(Oligodendroglioma) OR TITLE-ABS(Glioblastoma) OR TITLE-ABS(Neuroepithelial tumor) OR TITLE-ABS(Astroblastoma) OR TITLE-ABS(Ganglioglioma) OR TITLE-ABS(Gangliocytoma) OR TITLE-ABS(Neurocytoma) OR TITLE-ABS(Ependymoma) OR TITLE-ABS(Choroid plexus tumor*) OR TITLE-ABS(Choroid plexus papilloma) OR TITLE-ABS(Choroid plexus carcinoma) OR TITLE-ABS(Medulloblastoma) OR TITLE-ABS(Teratoid tumor*) OR TITLE-ABS(rhabdoid tumor*) OR TITLE-ABS(Pineocytoma) OR TITLE-ABS(Pineal parenchymal tumor*) OR TITLE-ABS(Pineoblastoma) OR TITLE-ABS(Papillary tumor*) OR TITLE-ABS(Desmoplastic myxoid tumor*) OR TITLE-ABS(Schwannoma) OR TITLE-ABS(Neurofibroma) OR TITLE-ABS(Perineurioma) OR TITLE-ABS(Nerve sheath tumor) OR TITLE-ABS(Paraganglioma) OR TITLE-ABS(Meningioma) OR TITLE-ABS(Mesenchymal tumor*) OR TITLE-ABS(Fibroblastic tumor*) OR TITLE-ABS(myofibroblastic tumor) OR TITLE-ABS(Solitary fibrous tumor) OR TITLE-ABS(Vascular tumor*) OR TITLE-ABS(Hemangioma*) OR TITLE-ABS(vascular malformation*) OR TITLE-ABS(Hemangioblastoma) OR TITLE-ABS(Skeletal muscle tumor*) OR TITLE-ABS(Rhabdomyosarcoma) OR TITLE-ABS(Ewing sarcoma)

OR TITLE-ABS(Chondrosarcoma) OR TITLE-ABS(Melanocytic tumor*) OR TITLE-ABS(Hematolymphoid tumor*) OR TITLE-ABS(Lymphoma*) OR TITLE-ABS(Histiocytic tumor*) OR TITLE-ABS(Germ cell tumor*) OR TITLE-ABS(Teratoma) OR TITLE-ABS(Germinoma) OR TITLE-ABS(Embryonal carcinoma) OR TITLE-ABS(Yolk sac tumor) OR TITLE-ABS(Choriocarcinoma)

OR TITLE-ABS(sellar region tumor*) OR TITLE-ABS(Craniopharyngioma) OR TITLE-ABS(Pituicytoma) OR TITLE-ABS(Pituitary adenoma) OR TITLE-ABS(Pituitary blastoma) OR TITLE-ABS(Metastas*) OR TITLE-ABS(Anencephaly) OR TITLE-ABS(Cephalocele) OR TITLE-ABS(Spina bifida) OR TITLE-ABS(Arnold-Chiari malformation) OR TITLE-ABS(Congenital hydrocephalus) OR TITLE-ABS(Cerebral structural developmental anomal*) OR TITLE-ABS(Cerebellar structural developmental anomal*) OR TITLE-ABS(spinal cord developmental anomal*) OR TITLE-ABS(vertebral column developmental anomal*) OR INDEXTERMS(Nervous System Diseases) OR INDEXTERMS(Alzheimer Vaccines) OR INDEXTERMS(Myofibrillar myopathy) OR INDEXTERMS(Worster Drought syndrome) OR INDEXTERMS(Glioma) OR INDEXTERMS(Neoplasms Neuroepithelial) OR INDEXTERMS(Teratoma) OR INDEXTERMS(Neoplasms Nerve Tissue) OR INDEXTERMS(Hemangioma) OR INDEXTERMS(Myosarcoma) OR INDEXTERMS(Sarcoma Ewing) OR INDEXTERMS(Chondrosarcoma) OR INDEXTERMS(Neoplasms Germ Cell and Embryonal) OR INDEXTERMS(Craniopharyngioma)OR INDEXTERMS(neurologic disease) OR INDEXTERMS(autonomic dysfunction) OR INDEXTERMS(motor dysfunction) OR INDEXTERMS(cerebrovascular disease) OR INDEXTERMS(myotonia))

AND

(TITLE-ABS(Vaccin*) OR TITLE-ABS(immuniz*) OR TITLE-ABS(immunis*) OR INDEXTERMS(Vaccin*) OR INDEXTERMS(Immuniz*) OR INDEXTERMS(Immunis*))

S4. Quality appraisal

Country / Region [ref]	Scope and purpose	Stakeholder involvement	Rigour of development	Clarity of presentation	Applicability	Editorial independence	Overall quality	Overall assessment
Australia [1]	94.40%	83.30%	77.10%	94.40%	62.50%	58.30%	6	Recommended
Canada [2]	88.90%	77.80%	79.20%	94.40%	66.70%	91.70%	6	Recommended
New Zealand [3]	88.90%	50.00%	45.80%	94.40%	79.20%	8.30%	5	Recommended
Singapore [4]	83.30%	61.10%	50.00%	94.40%	75.00%	16.70%	5	Recommended
UK (Cerebral Palsy) [5]	100.00%	94.40%	97.90%	100.00%	95.80%	100.00%	7	Recommended
UK (Multiple Sclerosis) [6]	100.00%	94.40%	97.90%	100.00%	95.80%	100.00%	7	Recommended
Italy [7]	100.00%	94.40%	100.00%	94.40%	79.20%	75.00%	6	Recommended
USA [8]	100.00%	83.30%	87.50%	100.00%	62.50%	91.70%	6	Recommended
France [9]	100.00%	72.20%	75.00%	94.40%	41.70%	75.00%	6	Recommended
Europe [10]	100.00%	88.90%	89.60%	94.40%	62.50%	75.00%	6	Recommended
Argentina [11]	94.40%	77.80%	81.30%	88.90%	45.80%	58.30%	6	Recommended
USA [12]	88.90%	83.30%	62.50%	88.90%	66.70%	75.00%	6	Recommended

(1) Australian Technical Advisory Group on Immunisation. Australian Immunisation Handbook. Canberra: Australian Government; 2024. https://immunisationhandbook.health.gov.au/

(2) Public Health Agency of Canada. Canadian Immunization Guide: Immunization of persons with chronic diseases. Ottawa; 2024. https://www.canada.ca/en/public-health/services/publications/healthy-living/canadian-immunization-guide-part-3-vaccination-specific-populations/page-7-immunization-persons-with-chronic-diseases.html

(3) Health New Zealand – Te Whatu Ora. Immunisation Handbook. Wellington; 2024. https://digitalnz.org/records/54387970

(4) Society of Infectious Diseases Singapore. Handbook on Adult Vaccination in Singapore. 4th ed. Singapore; 2023. https://new.cfps.org.sg/assets/Circulars/2023/SG-SID-001-Handbook-v15-Highres-bleed.pdf

(5) NICE. Cerebral palsy in adults. NG119. London; 2019. https://www.nice.org.uk/guidance/ng119

(6) NICE. Multiple sclerosis in adults: management. NG220. London; 2022. https://www.nice.org.uk/guidance/ng220

(7) Istituto Superiore di Sanità (ISS), Società Italiana di Neurologia (SIN). Diagnosis and treatment of multiple sclerosis in adults. Sistema Nazionale Linee Guida; 2022 Jul 18. Available from: https://www.iss.it/documents/20126/8331678/LG-340-SIN_SM.pdf/b3801eb6-2662-37b6-faeb-3612dd99364c?version=1.0&t=1677495451891

(8) Farez MF, Correale J, Armstrong MJ, et al. Practice guideline update summary: Vaccine-preventable infections and immunization in multiple sclerosis: Report of the Guideline Development, Dissemination, and Implementation Subcommittee of the American Academy of Neurology. Neurology. 2019;93(13):584-594. doi:10.1212/WNL.0000000000008157

(9) Lebrun-Frenay C, Carra-Dallière C, Louapre C, Papeix C, Pourcher V, Moisset X, et al; for the F4MS-SFSEP group. Recommandations de la Société francophone de la sclérose en plaques « Vaccinations et sclérose en plaques »: mise à jour 2024. *Pratique Neurologique – FMC*. 2024;15:105-113. doi:10.1016/j.praneu.2024.04.001

(10) Otero-Romero S, Lebrun-Frénay C, Reyes S, et al. ECTRIMS/EAN consensus on vaccination in people with multiple sclerosis: Improving immunization strategies in the era of highly active immunotherapeutic drugs. Mult Scler. 2023;29(8):904-925. doi:10.1177/13524585231168043

(11) Barboza AG, Sinay V, Silva BA, Rojas JI, Ysrraelit MC, Carnero Contentti E, et al. Guías de práctica para indicación y contraindicaciones de vacunación de pacientes con esclerosis múltiple. *Neurol Argent*. 2021;13(1):24-36. doi:10.1016/j.neuarg.2020.09.002

(12) Birnkrant DJ, Bushby K, Bann CM, et al. Diagnosis and management of Duchenne muscular dystrophy, part 3: primary care, emergency management, psychosocial care, and transitions of care across the lifespan. *Lancet Neurol*. 2018;17(5):445-455. doi:10.1016/S1474-4422(18)30026-7
